# Provident and Charitable Medical Assistance or State Medicine

**Published:** 1909-04-03

**Authors:** 


### Public Health and Hygiene.

### PROVIDENT AND CHARITABLE MEDICAL ASSISTANCE OR STATE MEDICINE.

At a recent meeting of the Council of the Poor-law Medical Officers' Association there was a unanimous agreement on several points of interest dealt with by the Royal Commission on the Poor Laws. These may be summarised as follows:—

1. The value of the past services of Poor-law medical officers and the recognition of the disadvantages under which they have laboured and the inadequacy of their remuneration.
2. The undesirability of organising medical assistance on a provident basis.
3. Disapprobation of the proposal to draft the State poor on to provident medical institutions.
4. Condemnation of the proposal to make every medical practitioner a Poor-law medical officer, or, as the Minority Report puts it, the supersession of the outdoor medical service of the Poor Law "by a publicly subsidised system of letting the poor choose their own doctor." And
5. Disapproval of the proposal to allow a poor person to claim State medical relief without the intervention of the relieving officer or assistance officer.

The Poor-law Medical Officers' Association must rejoice with other sections of the profession in the divergent reports in which the inquiries of the Royal Commission on the Poor Laws have culminated. Had the Commission been unanimous on the question of medical assistance and their unanimity taken the line of the Majority Report the interests of the medical profession would have been as directly threatened as the permanent solution of the problem of medical assistance of the poor would have been retarded.

The Council of the Poor-law Medical Officers' Association hold, and we think our readers will agree with them, that it would be futile and dangerous to attempt to reorganise Poor-law medical relief upon a provident basis.

We think that in the existing order of things the provident dispensary has its place, a great and important place, in the provision of medical attendance for the class unable to pay medical fees, but it is chimerical to suppose that provident institutions can be extended so as to embrace the large and unfortunate class with which the now discredited and certainly inadequate machinery of the Poor Law has hitherto dealt. A provident basis of organisation for a class which by nature, tradition, and the exigencies of its lot is essentially improvident, on the face of it is not promising. It is clear that whatever such organisation may be in name it will, in fact, rest upon quite other than a provident basis. Either it will be sustained by charity or by the State, or it will not be sustained at all. It is very important that the medical profession should face the situation quite frankly. Will the charitable contributions of the well-to-do compensate for the poverty and improvidence of the poorest classes in the community in the matter of providing medical relief? We have no hesitation in saying that they will not. The medical profession is groaning under the evils of the system of charitable provision at the present time; its burden will be unbearable should the system be extended so as to cover the whole ground of medical care and attention not directly paid for by the immediate recipients.

Not only will medical men be expected normally to contribute in services altogether disproportionately to their means, but when the resources of charity are inadequate, as on not infrequent occasions they will be, the claims of suffering humanity will continue to be met by the unfailing compassion of a profession which will not harden its heart at the importunate dictates of mere self-interest. The primal fact to be recognised is the catholicity of medicine. The one passport to its services should be the claim that they are needed. A fractured leg is to be set because the bone is broken and not because the patient can pay for treatment or has been provident enough to insure payment, or because the appeals of the injury to the charitable have brought it within the pale of provided relief. And the publio conscience can only properly be satisfied when it has been made a public charge to secure medical care whenever and wherever it may be needed and irrespective of the virtues or vices or status or resources of the recipients. As is clearly stated in the Minority Report, this does not necessarily involve the gratuitous provision of medical treatment to all applicants. But it does mean that the chargeability relating to the treatment of disease is a public responsibility and not a private concern of the medical practitioner who is trained to practise medicine and not charity or provident organisation. We are equally in agreement with the Poor-law Medical Officers' Association in their opposition to a publicly subsidised system of letting the poor choose their own doctor. As stated in the Minority Report, any such system would lead to an extravagant expenditure of public funds unchecked by official supervision and with no security for the due observance of hygienic requirements and of the conditions on which restoration to a healthy life is possible. A unified medical service will alone meet the circumstances of the case, and in such a unification of the public civil medical services lies the solution of the problem of dealing with medical attendance on the indigent and improvident, without injury to public or professional interests. We only differ from the Poor-law Medical Officers' Association in their desire to retain the relieving officer in his present position. We repeat that it is irrational to place any bar upon the rendering of medical services where they are needed. Where the aid of medicine is required let it be sought untramelled; but by the public organisation of chargeability let monetary interests be safeguarded by officials who are not medical men and who will not stand between medical men and their patients, but will see that services which have been properly rendered under State provisions are paid for as the equity of the circumstances demands. In other words, if we are to have relieving or assistance officers their function will not be such as their name implies, but the safeguarding of the public interest of chargeability. It is obvious that medicine will lose nothing, while the public gain will be great if there is provided a unified civil medical service available for all, but safeguarded from abuse. The recovery of such charges is a public, not a medical concern.

